# Association between socioeconomic status and prolonged television viewing time in a general Japanese population: NIPPON DATA2010

**DOI:** 10.1186/s12199-021-00978-6

**Published:** 2021-05-07

**Authors:** Yuka Sumimoto, Masahiko Yanagita, Naomi Miyamatsu, Nagako Okuda, Nobuo Nishi, Yosikazu Nakamura, Koshi Nakamura, Naoko Miyagawa, Motohiko Miyachi, Aya Kadota, Takayoshi Ohkubo, Tomonori Okamura, Hirotsugu Ueshima, Akira Okayama, Katsuyuki Miura, for NIPPON DATA2010 Research Group

**Affiliations:** 1 Department of Health and Sports Science, Doshisha University, 1-3 Tatara Miyakodani, Kyotanabe, Kyoto, 610-0394 Japan; 2 Department of Clinical Nursing, Shiga University of Medical Science, Tsukinowa-cho, Seta, Otsu, Shiga, 520-2192 Japan; 3 Department of Health and Nutrition, University of Human Arts and Sciences, 1288 Umagome, Iwatsuki-ku, Saitama, 339-8539 Japan; 4 International Center for Nutrition and Information, National Institute of Health and Nutrition, National Institutes of Biomedical Innovation, Health and Nutrition, 1-23-1 Toyama, Shinjuku, Tokyo, 162-8636 Japan; 5 Department of Public Health, Jichi Medical University, 3311-1 Yakushiji, Shimotsuke, Tochigi, 329-0498 Japan; 6 Department of Public Health and Hygiene, University of the Ryukyus, 207 Uehara, Nishihara, Okinawa, 903-0215 Japan; 7 Department of Preventive Medicine and Public Health, Keio University, 35 Shinanomachi, Shinjuku, Tokyo, 160-0016 Japan; 8 Department of Physical Activity Research, National Institute of Health and Nutrition , National Institutes of Biomedical Innovation, Health and Nutrition, 1-23-1 Toyama, Shinjuku, Tokyo, 162-8636 Japan; 9 Department of Public Health, Shiga University of Medical Science, Tsukinowa-cho, Seta, Otsu, Shiga, 520-2192 Japan; 10 Center for Epidemiologic Research in Asia, Shiga University of Medical Science, Tsukinowa-cho, Seta, Otsu, Shiga, 520-2192 Japan; 11 Department of Hygiene and Public Health, Teikyo University, 2-11-1 Kaga Itabashi, Tokyo, 173-0003 Japan; 12 Research Institute of Strategy for Prevention, Shinkawa 1-3-9 Chuohku, Tokyo, 104-0033 Japan

**Keywords:** Viewing television time, Socioeconomic factor, Japan, NIPPON DATA2010

## Abstract

**Background:**

It has been pointed out that prolonged television (TV) viewing is one of the sedentary behaviors that is harmful to health; however, the association between socioeconomic status (SES) and prolonged TV viewing time has not been sufficiently investigated in Japan.

**Methods:**

The study population are the participants of NIPPON DATA2010, which is a prospective cohort study of the National Health and Nutrition Survey 2010 in Japan. They were residents in 300 randomly selected areas across Japan. This study included 2752 adults. SES was classified according to the employment status, educational attainment, living status, and equivalent household expenditure (EHE). Prolonged TV viewing time was defined as more than or equal to 4 h of TV viewing per day. Multivariable logistic regression analyses were conducted to examine the association of SES with prolonged TV viewing time.

**Results:**

The mean TV viewing time was 2.92 h in all participants. Of 2752 participants, 809 (29.4%) prolonged TV viewing, and the mean TV viewing time of them was 5.61 h. The mean TV viewing time in participants without prolonged TV viewing time was 1.81 h. The mean TV viewing time was prolonged as age classes increased and significantly longer in aged ≥60 years. Prolonged TV viewing time was associated with not working for all age classes and sexes. Only among women, education attainment and living status were also associated with prolonged TV viewing time. For education attainment, the lower the received years of education, the higher odds ratios (OR) of prolonged TV viewing time. For living status, in women aged <60 years, living with others had a significantly higher OR compared to living with spouse. On the other hand, in women aged ≥60 years, living alone had a significantly higher OR. EHE did not have any significant associations with prolonged TV viewing time.

**Conclusions:**

In a general Japanese population, it should be noted that the association between SES and prolonged TV viewing time differed by age and sex. Particularly, it must draw attention to the prolonged TV viewing in elderly. The intervention in order to shorten TV viewing time needs to consider these attributes.

**Supplementary Information:**

The online version contains supplementary material available at 10.1186/s12199-021-00978-6.

## Introduction

The concern with the potential adverse health effects associated with sedentary behavior has been growing in the past decades. In November 2020, the World Health Organization announced the guidelines “physical activity and sedentary behaviour”. For the first time, this guideline provided recommendations in the associations between sedentary behavior and health outcomes [1]. According to this, we require to limit the amount of time spent being sedentary such as viewing TV and replace sedentary time with physical activity [1].

The reason why humans spent sedentary behavior for approximately 60% of awaking time [2] is the progress of automation due to socioeconomic development in all aspects of life. Shibata et al. reported that Japanese elderly spent 8.8 h of sedentary behavior daily, of which TV viewing time accounted for the most frequent activity at 45.5% [3]. In other words, TV viewing time is the most common sedentary behavior, but it is likely to be easily replaced with other physical activity among sedentary behavior.

Regarding the association between TV viewing time and health, previous studies reported that long-term TV viewing decreased short-term memory and cognitive function in adults [4, 5], and increased depression and Alzheimer’s disease in elderly [6, 7]. A meta-analysis study indicated that the pooled relative risks per 2 h of TV viewing per day were 1.20 (95% confidence intervals (95% CI), 1.14–1.27) for type 2 diabetes and 1.15 (95% CI, 1.06–1.23) for cardiovascular disease [8]. In particular, it was indicated that there was a dose-response relationship between all-cause mortality and prolonged TV viewing time, and it was significantly higher at more than 4 h a day [9]. Furthermore, TV viewing time was reported that it had stronger association compared with total sitting time [10].

TV viewing time tends to increase as age rises; it has also showed that various factors such as lifestyle, environment, socioeconomic status (SES), and other factors affect prolonged TV viewing time [11]. Regarding SES, it was reported that low SES such as low level of education/income was associated with prolonged TV viewing time [12–14]. However, these previous studies have investigated from Western countries which have different cultures and social backgrounds, but there were few studies conducted in Japan.

Therefore, the present study aimed to investigate associations between SES and prolonged TV viewing time in a general Japanese population. We analyzed baseline data obtained for NIPPON DATA2010 study which included participants in the National Health and Nutrition Survey Japan in 2010.

## Methods

### Study population

A prospective cohort study on cardiovascular disease, the National Integrated Project for Prospective Observation of Non-communicable Disease And its Trends in the Aged 2010 (NIPPON DATA2010), was established in 2010 [15]. This study was performed using data from the National Health and Nutrition Survey in November 2010 (NHNS2010) and the Comprehensive Survey of Living Conditions in June 2010 (CSLS2010), which were conducted by the Ministry of Health, Labour and Welfare of Japan.

In November 2010, 8815 residents aged 1 year and older from 300 randomly selected districts throughout Japan participated in the dietary survey for NHNS2010. Among 7229 participants age 20 years and older, 3873 participants (1598 men and 2275 women) had a blood test of NHNS2010 and were invited to enroll in NIPPON DATA2010. A total of 2898 participants (1239 men and 1659 women; participant rate, 74.6%) agreed to participate in the baseline survey for NIPPON DATA2010. Trained interviewers obtained written informed consent from all participants before enrollment. Data obtained from NHNS2010 and CSLC2010 were merged with data from NIPPON DATA2010. The Institutional Review Board of Shiga University of Medical Science (No. 22–29, 2010) approved this study.

For this study, of the 2898 participants, 91 were excluded because it was not possible to merge data from NHNS2010 or CSLC2010 with NIPPON DATA2010 baseline data. Additionally, seven who were over 90 years old, 26 who were lacking main variables, and 22 who were lacking confounding variables were excluded. The remaining 2752 participants (1172 men and 1580 women) were included in the present study.

### TV viewing time

To evaluate physical activity time by intensity, questions were posed about number of hours per day spent in heavy activity, moderate activity, slight activity, viewing TV, other sedentary activities, and no activities (sleeping) in the baseline survey of NIPPON DATA2010; the interviewer ensured that the total time added up to 24 h.

We used “viewing TV” to examine the association between SES and prolonged TV viewing time. More than 4 h viewing TV was defined as prolonged TV viewing time. The reason for this definition was that some studies showed that viewing TV for more than 4 h causes significantly higher hazard risk of all-cause mortality [9, 10, 16].

### Socioeconomic status

Information on SES was collected using self-administered questionnaires for NHNS2010 (employment status), CSLC2010 (living status, monthly household expenditure of May 2010, number of family member, house ownership), and NIPPON DATA2010 (educational attainment). Equivalent household expenditure (EHE) was calculated as monthly household expenditure divided by the square root of the number of family members and categorized into tertiles. House ownership was used to adjust the EHE because in the CSLC questionnaire rent in non-house owners was taken into account as a part of expenditure, but mortgage payments for home owners was not.

SES was defined as follows: (1) employment status (working [including self-employed] or not working [including students and homemakers]); (2) educational attainment (high [13 years or over], middle [10–12 years], low [<10 years]); (3) living status (living with spouse [including spouse and other family members], living with others [family members excluding spouse, e.g., parents, siblings, children, grandchildren], living alone); (4) EHE (first tertile [less than 106,000 yen], second tertile [106,000 yen or more but less than 162,000 yen], third tertile [162,000 yen or more]).

### Lifestyle and other variables

Public health nurses collected information on alcohol drinking habit, smoking habit, past histories, and exercise habit using a standardized questionnaire in NHNS. Alcohol drinking habit, smoking habit, and exercise habit were obtained from NHNS2010. Past histories were obtained from CSLC and NHNS2010. In CSLC2010, participants were asked if they had sought medical care for stroke or myocardial infarction, and they were asked if they had ever been told by a doctor that they had stroke or myocardial infarction in NHNS2010. Participants who answered “yes” to these questions were defined as having past histories of stroke and/or myocardial infarction.

These were classified as follows: (1) alcohol drinking habit (current drinker, ex-drinker, or non-drinker); (2) smoking habit (current smoker, ex-smoker, or non-smoker); (3) past histories (have past history [have histories of stroke and/or myocardial infarction] or does not have past history [does not have history of stroke and myocardial infarction]); (4) exercise habit (have an exercise habit or does not have an exercise habit).

### Statistical analysis

Statistical analyses were performed for men/women and for those aged<60 years/aged ≥60 years, separately, because basic living practices, e.g., working or not working, would differ substantially by sex and by age groups. In addition, the age of retirement was usually set at 60 years of age for indefinite-term employees at most workplaces in Japan.

TV viewing time was evaluated in seven age classes (20–29, 30–39, 40–49, 50–59, 60–69, 70–79, and 80–89) for men and women separately. To evaluate the association of SES and prolonged TV viewing time, the odds ratios (OR) and 95% CIs for prolonged TV viewing time were calculated by multiple logistic regression analyses, using explanatory variables (employment status, educational attainment, living status, and EHE) and possible confounding factors (alcohol drinking habit, smoking habit, exercise habit, living status, and past histories). We used three models. Model l was adjusted for age. Model 2 was further adjusted for alcohol drinking habit, smoking habit, exercise habit, and past histories. For model 3, we put in all of the SES factors and confounding factors simultaneously. For analyses on EHE, we additionally adjusted for house ownership (owned or rented). *P*<0.05 was considered statically significant. All statistical analyses were performed using SPSS version 25 for Windows.

## Results

### Characteristics of participants

Table 1 shows the distribution of age, employment status, educational attainment, EHE, and other variables by sex and age groups. The mean age of participants was 44.1 years in men aged<60 years, 70.2 years in men aged ≥ 60 years, 43.8 years in women aged<60 years, and 70.5 years in women aged ≥ 60 years. The percentages of working participants were higher in men (93.1% in aged < 60 years, 64.1% in aged ≥ 60 years) than in women (44.1% in aged < 60 years, 22.1% in aged ≥ 60 years) in both age groups. The percentage of participants with low level of education (< 10 years) was lower in aged <60 years (7.1% in men, 7.0% in women) than in aged ≥ 60 years (36.7% in men, 39.1% in women). In addition, instrumental activity of daily living (IADL) and current medical histories (hypertension, hypercholesterolemia, and diabetes mellitus) as assessed by blood test, blood pressure measurements, and use of medications are shown in Supplementary Table 1. IADL scores did not differ between age groups in both men and women, and the percentage of participants with current medical histories was higher in those participants in aged ≥ 60.

**Table 1:** Characteristics of study participants by sex and age groups, NIPPON DATA2010, 2010, Japan ( *n*=2749 )

	Men ( *n*=1,172 )			Women ( *n*=1,577 )		
	<60 years	≥60years	*P* value	<60 years	≥60years	*P* value
Number	463 (40)	709 (60.5)		740 (46.9)	837 (53.1)	
Age, years (SD)	44.1 (10.6)	70.2 (7.0)	<0.001	43.8 (10.4)	70.5 (7.0)	<0.001
Body mass index, kg/m^2^ (SD)	24.1 (3.6)	23.8 (2.9)	0.074	22.0 (3.6)	23.3 (3.4)	<0.001
Television viewing time, hour (SD)	2.4 (1.7)	3.8 (2.6)	<0.001	2.1 (1.8)	3.2 (2.2)	<0.001
Employment status, *n* (%)						
Working	431 (93.1)	313 (44.1)	<0.001	474 (64.1)	185 (22.1)	<0.001
Not working	32 (6.9)	396 (55.9)		266 (35.9)	652 (77.9)	
Educational attainment, *n* (%)						
High (13 ≤)	221 (47.7)	162 (22.8)	<0.001	378 (51.1)	100 (11.9)	<0.001
Middle (10–12)	209 (45.1)	287 (40.5)		310 (41.9)	410 (49.0)	
Low (<10)	33 (7.1)	260 (36.7)		52 (7.0)	327 (39.1)	
Living status, *n* (%)						
Living with spouse	333 (71.9)	601 (84.8)	<0.001	563 (76.1)	535 (63.9)	<0.001
Living with others	79 (17.1)	34 (4.8)		145 (19.6)	130 (15.5)	
Living alone	51 (11.0)	74 (10.4)		32 (4.3)	172 (20.5)	
Equivalent household expenditure, 10^4^JPY/month, *n* (%)						
1st tertile (< 10.6)	170 (36.7)	208 (29.3)	0.030	210 (28.4)	279 (33.3)	0.105
2nd tertile (10.6 - 16.2)	156 (33.7)	262 (37.0)		272 (36.8)	286 (34.2)	
3rd tertile (16.3≦)	137 (29.6)	239 (33.7)		258 (34.9)	272 (32.5)	
Smoking habit, *n* (%)						
Currently	177 (38.2)	141 (19.9)	<0.001	84 (11.3)	16 (1.9)	<0.001
Past	128 (27.6)	318 (44.9)		62 (8.4)	33 (3.9)	
Never	158 (34.1)	250 (35.3)		594 (80.3)	788 (94.2)	
Drinking alcohol habit, *n* (%)						
Currently	343 (74.1)	504 (71.1)	0.010	350 (47.3)	218 (26.0)	<0.001
Past	6 (1.3)	32 (4.5)		12 (1.6)	8 (1.0)	
Never	114 (24.6)	173 (24.4)		378 (51.1)	611 (73.0)	
Exercise habits, *n* (%)						
Have exercise habits	119 (25.7)	324 (45.7)	<0.001	168 (22.7)	328 (39.2)	<0.001
Not have exercise habits	344 (74.3)	385 (54.3)		572 (77.3)	509 (60.9)	
House ownership, *n* (%)						
Own house	347 (74.9)	606 (85.5)	<0.001	556 (75.1)	727 (86.9)	<0.001
Rented house	116 (25.1)	103 (14.5)		184 (24.9)	110 (13.1)	
Past of history (stroke, myocardial infarction) , *n* (%)						
Have past history	17 (3.7)	105 (14.8)	<0.001	3 (0.4)	72 (8.6)	<0.001
Not have past history	446 (96.3)	604 (85.2)		737 (99.6)	765 (91.4)	

*SD*, standard deviation; *JPY*, Japanese Yen

Data are presented as mean (SD) or as a number (%)

*P* value for differences between <60 years and ≥60 years were estimated by *t*-test for continuous variables, Mann-Whitney tests for television viewing time, and chi-squared tests for categorical variables

### Distribution of television viewing time

Table 2 shows the mean of TV viewing time for men and women in seven age classes. The mean TV viewing time was 2.92 h in all participants. Among the seven age classes, the mean TV viewing time was lowest in aged 30–39 years in men (2.00 h) and women (1.75 h). The mean TV viewing time increased as age classes increased, and it was highest in aged 80–89 years (4.49 h in men, 3.58 h in women).

**Table 2:** Mean TV viewing time by sex and age classes

	Total				< 4 h/day				≥ 4 h/day				*P* value^a^
	*n*	(%)	Mean (h/day)	(95% CI)	*n*	(%)	Mean (h/day)	(95% CI)	*n*	(%)	Mean (h/day)	(95% CI)	
Total													
	2749		2.92	(2.84, 3.01)	1940	(70.6)	1.81	(1.76, 1.85)	809	(29.4)	5.61	(5.47, 5.75)	<0.001
Men													
20–29 years	52	(4.4)	2.24	(1.73, 2.75)	39	(75.0)	1.40	(1.01, 1.78)	13	(25.0)	4.77	(4.25, 5.29)	<0.001
30–39 years	104	(8.9)	2.00	(1.73, 2.26)	88	(84.6)	1.57	(1.36, 1.78)	16	(15.4)	4.34	(4.03, 4.66)	<0.001
40–49 years	124	(10.6)	2.26	(1.99, 2.53)	98	(79.0)	1.67	(1.46, 1.89)	26	(21.0)	4.48	(4.22, 4.75)	<0.001
50–59 years	183	(15.6)	2.66	(2.37, 2.95)	140	(56.5)	1.88	(1.72, 2.04)	43	(23.5)	5.20	(4.52, 5.88)	<0.001
60–69 years	358	(30.5)	3.50	(3.24, 3.77)	222	(62.0)	1.99	(1.85, 2.13)	136	(38.0)	5.97	(5.59, 6.36)	<0.001
70–79 years	265	(22.6)	3.89	(3.59, 4.19)	141	(53.2)	2.10	(1.93, 2.27)	124	(46.8)	5.92	(5.55, 6.29)	<0.001
80–89 years	86	(7.3)	4.49	(3.81, 5.18)	40	(46.5)	1.76	(1.39, 2.14)	46	(53.5)	6.87	(6.16, 7.58)	<0.001
Women													
20–29 years	65	(4.1)	2.43	(1.85, 3.00)	50	(76.9)	1.44	(1.13, 1.74)	15	(23.1)	5.73	(4.46, 7.01)	<0.001
30–39 years	227	(14.4)	1.75	(1.55, 1.95)	200	(88.1)	1.36	(1.20, 1.51)	27	(11.9)	4.69	(4.44, 4.93)	<0.001
40–49 years	178	(11.3)	2.02	(1.74, 2.30)*	159	(89.3)	1.56	(1.39, 1.73)	19	(10.7)	5.84	(4.56, 7.13)	<0.001
50-59 years	270	(17.2)	2.42	(2.23, 2.62)	219	(81.1)	1.82	(1.68, 1.95)	51	(18.9)	5.02	(4.66, 5.38)	<0.001
60–69 years	415	(26.3)	3.07	(2.87, 3.27)*	285	(68.7)	1.98	(1.85, 2.10)	130	(31.3)	5.47	(5.16, 5.78)	<0.001
70–79 years	319	(20.3)	3.34	(3.09, 3.59)*	199	(62.4)	2.02	(1.86, 2.17)	120	(37.6)	5.54	(5.18, 5.90)	<0.001
80–89 years	103	(6.5)	3.58	(3.12, 4.05)	60	(58.3)	2.02	(1.73, 2.30)	43	(41.7)	5.77	(5.18, 6.36)	<0.001

*TV*, television; *CI*, confidence intervals

^a^*P* value for differences between <4 h/day and ≥4 h/day were estimated by Mann-Whitney tests

*P<0.05 significantly different between men and women using Mann-Whitney tests

Of 2752 participants, 809 (29.4%) prolonged TV viewing, the mean TV viewing time of them was 5.61 h. On the other hand, the mean TV viewing time in participants who did not prolong TV viewing time was 1.81h. There was a significant difference in TV viewing time between those with prolonged TV viewing time and those without it. The proportion of participants who prolonged TV viewing was increased as age classes increased in both men and women. The mean of physical activity time by intensity, excluding TV viewing time, is shown in Supplement Table 2.

### The association between SES and prolonged TV viewing time

Tables 3 and 4 show results from multiple logistic regression analysis using prolonged TV viewing time as an objective variable and socioeconomic factors as explanatory variables in men and women by age category. In model 3, significantly increased ORs for prolonged TV viewing time were observed for not working participants compared with working participants in all strata (OR 3.37 in men aged<60 years, 3.77 in women aged<60 years, 4.77 in men aged ≥60 years, 4.21 in women aged ≥ 60 years). However, educational attainment and living status were significantly associated with prolonged TV viewing time only among women. For education attainment, it was observed that the shorter the received years of education, the higher the ORs of prolonged TV viewing time (low, OR 2.63 in aged<60 years, OR 2.34 in aged≥60 years; middle, OR 1.72 in aged<60 years, OR 2.00 in aged≥60 years). For living status, the association between prolonged TV viewing time and living status differed by age category among women. In women aged<60 years, living with others was significantly associated with prolonged TV viewing time compared to living with spouse (OR 1.95). On the other hand, in women aged≥60 years, living alone was significantly associated with prolonged TV viewing time (OR 1.84). Regarding EHE, it showed no significant associations with prolonged TV viewing time in all strata. These results were similar even after adjusting for BMI and IADL scores (data not shown).

**Table 3:** Association between socioeconomic status and prolonged TV viewing time in men (*n*=1172): NIPPON DATA2010

	*n*	%^a^	Model 1		Model 2		Model 3	
			OR	95% CI	OR	95% CI	OR	95% CI
**<60 years**								
Employment status								
Working	431	19.3		(ref.)		(ref.)		(ref.)
Not working	32	46.9	3.68	(1.76–7.68)	3.76	(1.74–8.12)	3.37	(1.50–7.56)
Educational attainment								
High (13≤ )	221	14.0		(ref.)		(ref.)		(ref.)
Middle (10–12)	209	28.2	2.39	(1.47–3.89)	2.36	(1.44–3.87)	2.37	(1.43–3.92)
Low (<10)	33	24.2	1.90	(0.78–4.67)	1.67	(0.66–4.22)	1.27	(0.47–3.44)
Living status								
Living with spouse	333	19.2		(ref.)		(ref.)		(ref.)
Living with others	79	22.8	1.41	(0.75–2.67)	1.35	(0.70–2.58)	1.18	(0.58–2.37)
Living alone	51	31.4	1.97	(1.03–3.79)	1.98	(1.02–3.85)	1.96	(0.94–4.10)
Equivalent household expenditure								
1st tertile	170	22.4		(ref.)		(ref.)		(ref.)
2nd tertile	156	20.5	0.88	(0.52–1.50)	0.94	(0.55–1.61)	0.93	(0.53–1.63)
3rd tertile	137	20.4	0.88	(0.51–1.53)	0.89	(0.51–1.56)	0.87	(0.48–1.56)
**≥ 60 years**								
Employment status								
Working	313	24.0		(ref.)		(ref.)		(ref.)
Not working	396	58.3	4.44	(3.13–6.31)	4.75	(3.31–6.82)	4.77	(3.31–6.88)
Educational attainment								
High (13≤)	162	39.5		(ref.)		(ref.)		(ref.)
Middle (10–12)	287	43.2	1.22	(0.82–1.81)	1.20	(0.81–1.79)	1.24	(0.81–1.90)
Low (<10)	260	45.4	1.19	(0.80–1.79)	1.17	(0.78–1.75)	1.26	(0.80–1.96)
Living status								
Living with spouse	601	41.1		(ref.)		(ref.)		(ref.)
Living with others	34	61.8	2.40	(1.17–4.93)	2.38	(1.16–4.90)	1.86	(0.87–3.98)
Living alone	74	51.4	1.56	(0.96–2.54)	1.54	(0.94–2.52)	1.20	(0.69–2.08)
Equivalent household expenditure								
1st tertile	208	45.2		(ref.)		(ref.)		(ref.)
2nd tertile	262	43.1	0.91	(0.63–1.32)	0.92	(0.64–1.34)	0.87	(0.58–1.30)
3rd tertile	239	41.4	0.86	(0.59–1.26)	0.88	(0.60–1.29)	0.85	(0.56–1.30)

*TV*, television; *OR*, odds ratio; *CI*, confidence intervals

^a^Proportion of defined as participants who viewed TV over 4 h

Model l was adjusted for age for each socioeconomic factor

Model 2 was adjusted for model 1 plus alcohol drinking habit, smoking habit, exercise habit, and past history

Model 3 was adjusted for all socioeconomic factor (employment status, educational attainment, living status, equivalent household expenditure), age, alcohol drinking habit, smoking habit, exercise habit, and past history, simultaneously

**Table 4:** Association between socioeconomic status and prolonged TV viewing time in women (*n*=1577): NIPPONDATA2010

			Model 1		Model 2		Model 3	
	*n*	%^a^	OR	95%CI	OR	95%CI	OR	95%CI
**<60 years**								
Employment status								
Working	474	9.3		(ref.)		(ref.)		(ref.)
Not working	266	25.6	3.38	(2.23–5.12)	3.35	(2.20–5.10)	3.77	(2.43–5.84)
Educational attainment								
High (13≤)	378	11.1		(ref.)		(ref.)		(ref.)
Middle (10–12)	310	18.1	1.72	(1.12–2.66)	1.65	(1.06–2.57)	1.72	(1.09–2.71)
Low (<10)	52	26.9	2.84	(1.41–5.70)	2.69	(1.31–5.52)	2.63	(1.21–5.69)
Living status								
Living with spouse	563	14.6		(ref.)		(ref.)		(ref.)
Living with others	145	17.1	1.40	(0.83–2.34)	1.39	(0.83–2.34)	1.95	(1.11–3.41)
Living alone	32	15.6	1.05	(0.39–2.82)	1.07	(0.40–2.91)	1.26	(0.43–3.68)
Equivalent household expenditure								
1st tertile	210	14.8		(ref.)		(ref.)		(ref.)
2nd tertile	272	17.6	1.20	(0.73–1.96)	1.24	(0.75–2.05)	1.46	(0.86–2.47)
3rd tertile	258	12.8	0.81	(0.48–1.38)	0.83	(0.48–1.42)	0.89	(0.50–1.56)
**≥60 years**								
Employment status								
Working	185	14.6		(ref.)		(ref.)		(ref.)
Not working	652	40.8	3.93	(2.51–6.13)	3.88	(2.48–6.09)	4.21	(2.65–6.70)
Educational attainment								
High (13≤)	100	20.0		(ref.)		(ref.)		(ref.)
Middle (10–12)	410	34.6	2.08	(1.22–3.54)	2.13	(1.25–3.65)	2.00	(1.15–3.49)
Low (<10)	327	40.1	2.49	(1.44–4.28)	2.61	(1.50–4.52)	2.34	(1.32–4.16)
Living status								
Living with spouse	535	31.0		(ref.)		(ref.)		(ref.)
Living with others	130	33.8	1.06	(0.70–1.62)	1.10	(0.72–1.67)	1.07	(0.68–1.67)
Living alone	172	48.3	1.90	(1.31–2.76)	1.89	(1.30–2.77)	1.84	(1.22–2.75)
Equivalent household expenditure								
1st tertile	279	38.0		(ref.)		(ref.)		(ref.)
2nd tertile	286	32.9	0.82	(0.57–1.16)	0.79	(0.56–1.13)	0.72	(0.49–1.05)
3rd tertile	272	34.2	0.87	(0.61–1.23)	0.85	(0.59–1.21)	0.83	(0.57–1.22)

*TV*, television; *OR*, odds ratio; *CI*, confidence intervals

^a^Proportion of defined as participants who viewed TV over 4 h

Model l was adjusted for age for each socioeconomic factor

Model 2 was adjusted for model 1 plus alcohol drinking habit, smoking habit, exercise habit, and past history

Model 3 was adjusted for all socioeconomic factor (employment status, educational attainment, living status, equivalent household expenditure), age, alcohol drinking habit, smoking habit, exercise habit, and past history, simultaneously

## Discussion

In the present analysis of a nationwide cross-section study of a randomly selected sample of adults in a Japanese population, it was observed that TV viewing time was longer as age rose and significantly longer in men than in women among elderly. Regarding the association between SES and prolonged TV viewing time, not working was related to prolonged TV viewing time in all ages and sexes. In women, education attainment and living status were related to prolonged TV viewing time.

We found that TV viewing time was longer as age rose, and this finding is consistent with a previous study: Shields and Tremblay reported TV viewing time by age classes, and TV viewing time was longer as age rose [17]. In recent years, the younger generation use computer and mobile phone due to the expansion of the Internet and tend to have shorter viewing TV time [18]. However, the length of TV viewing time for the elderly has not changed significantly [18], and it can be said that prolonged TV viewing has become a habit. That is, prolonged TV viewing is a serious problem for the elderly.

Regarding the association between SES and prolonged TV viewing time, for employment status, we found that not working had an association with prolonged TV viewing time, and this finding supported the results of previous studies [12–14, 19]. Particularly, TV viewing time in retirees was more prolonged [20–22]. Accordingly, it seems to be that retirement is an important transition to change lifestyle habits. We suggest that intervention in order to shorten TV viewing time may be effective before/after retirement.

For education attainment, previous studies reported that education attainment had a negative association with prolonged TV viewing time [12, 23, 24]. In general, education is the portal to occupational option and higher income and can affect health behavior [25]. Persons who think cost is a barrier to being active were likely to prolong TV time [26]. Viewed in this light, lower education persons have lower income and health consciousness and are likely to choose TV as cheap and popular entertainment. In this study, only women supported these results of previous studies. It was indicated that the association between education attainment and prolonged TV viewing time differed by sex in Japan.

For living status, we found that not living with spouse had a significantly higher risk of prolonged TV viewing in only women. Previous studies reported that living alone or being unmarried was associated with prolonged TV viewing [12, 14]. In women aged<60 years, the reason for prolonged TV viewing time is because the length of free time differed by living status. In fact, married women have less free time as their life stage progresses and their role increases [27]. On the other hand, in women aged≥60years, it may be that there were some reasons for prolonged TV viewing time. First, a previous study reported that the rate of driver’s license was lower in elderly women; elderly women living alone stayed home to limit transportation and tended to prolong TV viewing time [12]. Second, approximately 80% of elderly answered that TV was not only daily enjoyment but also an information tool [28]. Therefore, it can be said that TV viewing naturally blended into their daily life. For the above reasons, elderly women living alone may need to consciously shorten TV viewing time. There are no previous studies from Asian or Western countries that have examined the association between living status and prolonged TV viewing time, stratified by sex and age groups. Therefore, it was not clear whether the result of this study could be a characteristic of Japanese. However, the results of this study are consistent with a previous study from Japan [12] and should be clarified by further studies.

This study has some strength. First, it is reliable for evaluating physical activity. Self-administered questionnaires and interviews were also conducted by well-trained researchers. Therefore, daily TV viewing hours were obtained accurately. Second, smartphones have become widespread in Japan since 2010; the data of this study were not yet affected by smartphones.

This study has also some limitations. Because of the cross-sectional nature of this study, we were unable to determine whether there was a causal association between SES and prolonged TV viewing time. Second, in this study, TV viewing time does not include using a computer or playing games; the definition of screen time in previous studies from other countries may be slightly different. This difference may not affect older people, but sedentary time in young people may be undervalued due to the fact that using a computer and playing game are not included. Finally, the participation rate of NHNS2010 participants in NIPPON DATAD2010 (75%) was relatively high, but the participation rate for blood tests was low at 54%. Therefore, it is possible that the target population is biased toward those who are health conscious and cooperative.

## Conclusion

The mean TV viewing time was prolonged as age rose and was significantly longer in men than in women among elderly. Particularly, elderly need to consciously shorten TV viewing time. Employment status was associated with prolonged TV viewing time regardless of sex and age. However, there were gender differences for education attainment and living status. The intervention in order to shorten TV viewing time needs to consider these attributes into account.

## Supplementary Information

**Additional file 1: Supplement Table 1**. Participants’ instrumental activity of daily living and current medical histories by sex and age groups. **Supplement Table 2**. Mean physical activities time by intensity in sex and age classes. **Supplement Table 3**. Mean TV viewing time by socioeconomic status.

## Data Availability

The datasets are not open to the public.

## Abbreviations

95% CIs: 95% confidence intervals
CSLS: Comprehensive Survey of Living Conditions
EHE: Equivalent household expenditure
NHNS: National Health and Nutrition Survey
NIPPON DATA: National Integrated Project for Prospective Observation of Non-communicable Disease And its Trends in the Aged
OR: Odds
SES: Socioeconomic status
TV: Television

## References

[1] World Health Organization, WHO guidelines on physical activity and sedentary behaviour, 2020. Published 2020. Available from: https://www.who.int/publications/i/item/9789240015128. Accessed 10 Jan 2021.

[2] Bauman A, Ainsworth BE, Sallis JF, Hagströmer M, Craig CL, Bull FC, Pratt M, Venugopal K, Chau J, Sjöström M, IPS Group (2011). The descriptive epidemiology of sitting. A 20-country comparison using the International Physical Activity Questionnaire (IPAQ). Am J Prev Med..

[3] Shibata A, Oka K, Ishii K, Miyawaki R, Inoue S, Sugiyama T, Owen N (2019). Objectively-assessed patterns and reported domains of sedentary behavior among Japanese older adults. J Epidemiol..

[4] Bakrania K, Edwardson CL, Khunti K, Bandelow S, Davies MJ, Yates T (2018). Associations between sedentary behaviors and cognitive function: cross-sectional and prospective findings from the UK Biobank. Am J Epidemiol..

[5] Fancourt D, Steptoe A (2019). Television viewing and cognitive decline in older age: findings from the English Longitudinal Study of Ageing. Sci Rep..

[6] Werneck AO, Oyeyemi AL, Szwarcwald CL, Vancampfort D, Silva DR (2018). Associations between TV viewing and depressive symptoms among 60,202 Brazilian adults: the Brazilian national health survey. J Affect Disord..

[7] Hamer M, Stamatakis E (2014). Prospective study of sedentary behavior, risk of depression, and cognitive impairment. Med Sci Sports Exerc..

[8] Grøntved A, Hu FB (2011). Television viewing and risk of type 2 diabetes, cardiovascular disease, and all-cause mortality: a meta-analysis. JAMA..

[9] Dunstan DW, Barr EL, Healy GN, Salmon J, Shaw JE, Balkau B (2010). Television viewing time and mortality: the Australian Diabetes, Obesity and Lifestyle Study (AusDiab). Circulation..

[10] Patterson R, McNamara E, Tainio M, de Sá TH, Smith AD, Sharp SJ, Edwards P, Woodcock J, Brage S, Wijndaele K (2018). Sedentary behaviour and risk of all-cause, cardiovascular and cancer mortality, and incident type 2 diabetes: a systematic review and dose response meta-analysis. Eur J Epidemiol..

[11] Rhodes RE, Mark RS, Temmel CP (2012). Adult sedentary behavior: a systematic review. Am J Prev Med..

[12] Kikuchi H, Inoue S, Sugiyama T, Owen N, Oka K, Shimomitsu T (2013). Correlates of prolonged television viewing time in older Japanese men and women. BMC Public Health.

[13] Bowman SA (2006). Television-viewing characteristics of adults: correlations to eating practices and overweight and health status. Prev Chronic Dis..

[14] Clark BK, Sugiyama T, Healy GN, Salmon J, Dunstan DW, Shaw JE, Zimmet PZ, Owen N (2010). Socio-demographic correlates of prolonged television viewing time in Australian men and women: the AusDiab study. J Phys Act Health..

[15] Kadota A, Okuda N, Ohkubo T, Okamura T, Nishi N, Ueshima H (2018). The National Integrated Project for Prospective Observation of Non-communicable Disease and its Trends in the Aged 2010 (NIPPON DATA2010): objectives, design, and population characteristics. J Epidemiol.

[16] Sun JW, Zhao LG, Yang Y, Ma X, Wang YY, Xiang YB (2015). Association between television viewing time and all-cause mortality: a meta-analysis of cohort studies. Am J Epidemiol..

[17] Shields M, Tremblay MS (2008). Screen time among Canadian adults: a profile. Health Rep..

[18] Ministry of Internal Affairs and Communications. 2019 WHITE PAPER Information and Communications in Japan, 2019. Available from: https://www.soumu.go.jp/johotsusintokei/whitepaper/eng/WP2019/chapter-1.pdf#page=1. Published 2019. Accessed 10 Jan 2021.

[19] Sugiyama T, Healy GN, Dunstan DW, Salmon J, Owen N (2008). Is television viewing time a marker of a broader pattern of sedentary behavior?. Ann Behav Med..

[20] Touvier M, Bertrais S, Charreire H, Vergnaud AC, Hercberg S, Oppert JM (2010). Changes in leisure-time physical activity and sedentary behaviour at retirement: a prospective study in middle-aged French subjects. Int J Behav Nutr Phys Act.

[21] Sprod J, Ferrar K, Olds T, Maher C (2015). Changes in sedentary behaviours across the retirement transition: a systematic review. Age Ageing..

[22] Flood SM, Moen P (2015). Healthy time use in the encore years: do work, resources, relations, and gender matter?. J Health Soc Behav..

[23] De Cocker K, De Bourdeaudhuij I, Teychenne M, McNaughton S, Salmon J (2013). Educational inequalities in TV viewing among older adults: a mediation analysis of ecological factors. Int J Behav Nutr Phys Act.

[24] Ding D, Sugiyama T, Winkler E, Cerin E, Wijndaele K, Owen N (2012). Correlates of change in adults’ television viewing time: a four-year follow-up study. Med Sci Sports Exerc..

[25] Glymour MM, Avendano M, Kawachi I. Socioeconomic status and health. Social Epidemiology Second Edition. New York: Oxford University Press; 2014. p. 17–62.

[26] Salmon J, Owen N, Crawford D, Bauman A, Sallis JF (2003). Physical activity and sedentary behavior: a population-based study of barriers, enjoyment, and preference. Health Psychol..

[27] Statistics Bureau of Japan. Summary of the results, survey on time use and leisure activities in Japan, 2012. (in Japanese). Available from: https://www.stat.go.jp/data/shakai/2011/pdf/gaiyou2.pdf. Published 2012. Accessed 10 Jan 2021.

[28] Cabinet Office. Survey on the consciousness of older people about daily life in Japan, 2014. (in Japanese). Available from: https://www8.cao.go.jp/kourei/ishiki/h26/sougou/zentai/index.html. Published 2014. Accessed 10 Jan 2021.

